# Cytomegalovirus Retinitis: The Neglected Disease of the AIDS Pandemic

**DOI:** 10.1371/journal.pmed.0040334

**Published:** 2007-12-01

**Authors:** David Heiden, Nathan Ford, David Wilson, William R Rodriguez, Todd Margolis, Bart Janssens, Martha Bedelu, Nini Tun, Eric Goemaere, Peter Saranchuk, Kalpana Sabapathy, Frank Smithuis, Emmanuel Luyirika, W. Lawrence Drew

## Abstract

The authors describe CMV retinitis in resource-poor settings and suggest possibilities for management.

Cytomegalovirus (CMV), a member of the herpesvirus family, was a familiar cause of blindness and death in patients with advanced AIDS in Western countries prior to the introduction of highly active antiretroviral therapy (HAART). CMV retinitis then occurred in roughly one-third of patients with AIDS, and accounted for over 90% of cases of HIV-related blindness [1]. Extraocular CMV disease was a major cause of AIDS-related morbidity and mortality [2]. CMV retinitis is now clinically infrequent in patients with AIDS in developed countries, thanks to the widespread availability of HAART, although the problem has not disappeared [3,4]. Successful fundamentals of management are screening eye examinations in patients with low CD4 counts, and effective anti-CMV treatment with ganciclovir and related compounds, combined with HAART.

In developing regions of the world where the HIV/AIDS pandemic is rapidly unfolding, CMV retinitis is a neglected disease, largely undiagnosed and untreated. Workable diagnostic and therapeutic strategies have not yet been defined, and CMV is absent from current and pending World Health Organization (WHO) guidelines for the management of HIV in resource-limited settings. Similarly, the WHO's ambitious “Vision 2020” program, which seeks to provide guidance on the use of ophthalmologic resources until the year 2020, fails to mention CMV retinitis.

The scale of the CMV problem in developing regions is still not known, as most cases of CMV retinitis are never diagnosed. Routine retinal examination is rarely performed and the diagnosis may not be considered unless a patient has damaged vision, or even becomes irreversibly blind. Mortality due to extraocular CMV disease is almost impossible to attribute without autopsy, and so is wrongly ascribed, usually to “advanced HIV infection” or tuberculosis. Yet it has been estimated that “between 5% and 25% of all HIV-infected patients in the developing world can be expected to develop this blinding disorder at some point during the course of their illness” [5], leading some to warn of a possible “epidemic of blindness” [6]. Recent direct evidence for the substantial scope of this neglected problem comes from a tertiary care ophthalmology center in Chang Mai, Thailand, where in a large consecutive series of referrals, 19% of the cases of bilateral blindness were caused by CMV retinitis, following only cataract as a cause of blindness, and exceeding glaucoma, age-related macular degeneration, and diabetic retinopathy [7].

In this article we provide preliminary data describing the problem and suggest possibilities for management of CMV retinitis in resource-poor settings. Our observations are based on the clinical experience from Médecins Sans Frontières (MSF) HIV/AIDS projects in Cambodia, South Africa, Lesotho, Myanmar, Thailand, and China, and on field assessments of four of these programs, and other programs at other locations, by the corresponding author (DH), an ophthalmologist with clinical training and experience with uveitis and CMV retinitis.

## Epidemiology

In developing countries CMV infection is usually acquired in childhood, and nearly 100% of adults are seropositive [8,9,10]. Like other herpesvirus infections, CMV may remain latent for life. Overt clinical disease occurs with waning immunity.

Available data on the epidemiology of CMV retinitis in developing countries are difficult to interpret, as the patients are often not stratified by CD4 lymphocyte counts, and both the technique and quality of retinal examination are variable. The “gold standard” for diagnosing CMV retinitis is indirect ophthalmoscopy with fully dilated pupils, yet some studies are done without dilation, and some use only the direct ophthalmoscope with a narrow field of view that will miss many cases of infection [11]. Although prevalence estimates for HIV infection are now generally available, the proportion of HIV-infected patients with advanced disease and CD4 counts below 50 cells/μl, the level of immunodeficiency at which CMV retinitis generally occurs, is often not known. As such, it is difficult to extrapolate the magnitude of the epidemic.

**Summary Points**

- In Western countries in the pre-HAART era, about 1/3 of patients with AIDS suffered potentially blinding CMV retinitis. Extraocular CMV infection in the CNS, gastrointestinal tract, and other organs contributed to AIDS-related mortality.
- In developing countries, CMV retinitis has been neglected, with little data describing the scope of the problem, and no strategy for management of the disease.
- Retinal screening examinations were conducted at the primary care level in AIDS clinics in five countries of sub-Saharan Africa and Southeast Asia in 325 patients with CD4 counts below 50 cells/μl. Twenty percent of patients had CMV retinitis, usually not previously diagnosed, and additional studies found 37% of individual eyes with CMV retinitis were blinded by the infection.
- Successful management of CMV retinitis is a realistic goal, and must begin with decentralizing diagnostic capacity to the primary care level. All high-risk patients (at minimum all patients with CD4 count below 50 cells/μl) must have retinal screening examination with the pupil fully dilated and using an indirect ophthalmoscope.
- Treatment with valganciclovir, an oral medication that is effective for both ocular and systemic disease, is essential. Valganciclovir, a single-source monopoly product, is prohibitively expensive at present, but must be made available and affordable.

From the limited data available, it appears that the extent of the problem in Southeast Asia is similar to that observed in the pre-HAART era in Europe and the United States. Ophthalmology centers in Chiang Mai, Thailand, and Chennai, India report prevalence rates of CMV retinitis in patients with HIV of 33% and 17%, respectively [12,13], although other Southeast Asian studies have reported lower figures [14].

In Africa, the CMV problem appears to be less severe, with reported prevalence rates from cross-sectional surveys ranging from 0%–8.5%. [15]. However, a longitudinal study from Togo, which followed 200 patients for 20 months, found a cumulative CMV retinitis incidence of 21.4% [16]. In that setting, mean survival after a diagnosis of CMV retinitis was 22 days. The short duration from diagnosis to death in this example suggests that cross-sectional surveys may underestimate the cumulative risk of CMV.

In resource-poor settings, underdiagnosis is clearly the most significant problem in establishing an accurate picture of the epidemiology of CMV. But several issues besides diagnostic constraints might explain discrepancies in reported prevalence of CMV retinitis. Plausible explanations notably include regional variation in the length of survival of patients with low CD4 counts—short survival times reducing the period of risk for CMV—and different genetically determined host vulnerabilities to CMV [17].

Our screening studies in patients with CD4 counts below 50 cells/μl (Table 1) show a high attack rate in Southeast Asia, and a lower prevalence in more limited studies from sub-Saharan Africa, where, nevertheless, CMV retinitis still represents a substantial problem due to the large absolute numbers of people infected with HIV. Our observations support the hypothesis that CMV retinitis is substantially underdiagnosed.

**Table 1:**
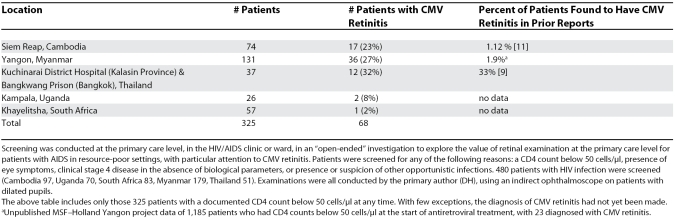
CMV Retinitis in Patients with AIDS with CD4 Counts Below 50 Cells/μl

## Diagnosis and Clinical Course of CMV Retinitis

The “gold standard” for diagnosis of CMV retinitis is examination of the retina through a dilated pupil by a skilled clinician using an indirect ophthalmoscope. Dilation of the pupil is critical, because CMV retinitis can occur anywhere in the retina and without a dilated pupil only a fraction of the retina can be examined. Retinal examination is highly sensitive and specific, and can be performed by any clinician after about one month's training. Diagnosis of CMV retinitis does not require laboratory tests or eye tests [18]. Patients are typically easy to examine because the ocular media is clear and the pupil dilates well. With a skilled examiner and a cooperative patient with dilated pupils, the exam can be performed in less than two minutes.

**Figure 1:**
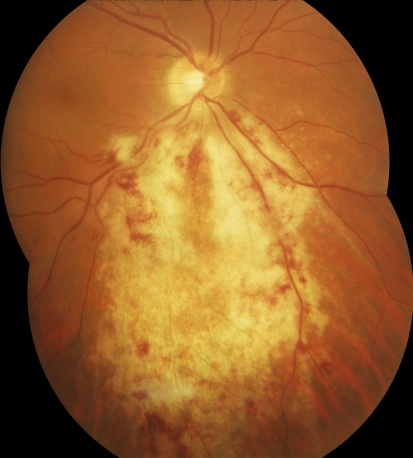
Example of CMV Retinitis

CMV retinitis is characterized by dense retinal whitening, which can vary in appearance from “fluffy” to “dry and granular.” Hemorrhage is frequently present, but in highly variable amounts, and may be absent. The retinitis tends to follow vessels, spreading centrifugally, often in a brush-fire fashion, with central clearing where the retina has already been completely destroyed; the border of active retinitis is irregular, and small white satellite lesions are highly characteristic (Figure 1).

In untreated patients with AIDS, the natural history of CMV retinitis is a slow but relentlessly progressive necrotizing retinitis with destruction of the entire retina in three to six months [19,20]. The zone of retinitis advances at a rate of approximately 750 microns, or half a “disc diameter” every three weeks. Based on the rate of spread, the most common condition confused with CMV retinitis—HIV retinopathy with “cotton-wool” spots—can easily be distinguished by repeat examination after three to four weeks. “Cotton-wool” spots condense or fade while CMV retinitis advances.

Blindness from CMV is permanent, and may occur well before full destruction of the retina. There are three mechanisms for vision loss, and for each mechanism early diagnosis is crucial to preserve vision. First, blindness may occur from direct damage to the macula or optic nerve. Second, blindness can result from retinal detachment, either during the acute infection or later, even years after the CMV retinitis has resolved. In the pre-HAART era in Western countries, patients with CMV retinitis developed retinal detachment at a rate of 33% per eye/per year [21]. In resource-poor settings, a patient who develops a CMV-related retinal detachment will be left with a permanently blind eye. Finally, up to 20% of patients with CMV retinitis may develop immune recovery uveitis (IRU) [22], after reconstitution of the immune system by HAART. IRU may cause vision loss from vitritis, retinal membrane formation, cystoid macular edema, or cataract. The larger the area of CMV retinitis, the greater the risk of both retinal detachment and IRU [23,24], again providing a compelling rationale for the benefit of early diagnosis and treatment.

## Symptoms and the Need for Systematic Retinal Screening

CMV retinitis does not cause pain or redness in the involved eye. Characteristic symptoms are floaters, scotoma, photopsia, and blurred vision, [12,25] and although some reports suggest symptoms are common [25], they are frequently discounted or ignored. Clinical experience shows that limiting retinal examination only to patients with symptoms is not reliable, and that systematic retinal screening examination of vulnerable patients with or without symptoms is essential [26]. The efficacy of screening asymptomatic patients has been demonstrated [27], and meets generally accepted criteria for appropriate screening interventions: the disease is common, treatable, and easy to diagnose at an early stage, and the consequence of blindness is severe. Determination of visual acuity with an eye chart, the most commonly used eye screening test, measures only foveal function, representing less than 1% of the retinal surface, and is a poor test for CMV retinitis.

We believe that systematic screening examination of the retina should become a fundamental requirement of HIV-related care in resource-poor settings. In our recent work in Myanmar, 13/42 (31%) patients diagnosed with CMV retinitis had no symptoms; an MSF study in Cambodia that used a questionnaire specifically designed to elicit symptoms of CMV retinitis found that almost half of the patients with CMV retinitis (17/39, 44%) had no symptoms at the time the disease was detected by screening examination [28].

## Treatment

Successful treatment of CMV in patients with AIDS requires both specific medication against CMV and recovery of immune function through the use of antiretroviral therapy. Antiretrovirals are continued indefinitely, while specific treatment for CMV retinitis is continued at least until the retinitis resolves. Once there is some restitution of immune function and the CD4 count is increased to above 100 cells/μl (and commonly after at least three months), reactivation of CMV retinitis is unlikely [29,30]. Thus specific treatment of CMV retinitis is usually required for only a limited vulnerable window of time.

Ganciclovir, the traditional “gold standard” for treatment of CMV [31,32], can be administered systemically (daily or twice daily intravenous infusion), or locally (intraocular injection). Valganciclovir, a well-absorbed valine ester prodrug of ganciclovir, can achieve equivalent blood levels when given by mouth, and is equally effective as intravenous ganciclovir [33]. Other anti-CMV agents such as foscarnet and cidofovir are more toxic or expensive, and no more effective [34,35].

At this time only intraocular ganciclovir injection—local treatment—is considered a viable option in resource-poor settings. For both intravenous ganciclovir and oral valganciclovir, the primary issue is drug cost. A two-weeks induction treatment with intravenous ganciclovir costs US$1,583, and oral valganciclovir costs US$2,136. For maintenance treatment intravenous ganciclovir costs US$57 each day, and oral valganciclovir costs US$76 each day, based on average wholesale price in the United States [36]. In contrast, the cost of the medication in a single weekly intraocular ganciclovir injection is only US$0.57. But these figures are misleading because they ignore the cost of surgical supplies (often not available), and do not put a dollar amount on physician time and skills.

Intraocular injection of ganciclovir is certainly preferable to no treatment, even though it fails to address systemic disease. This treatment is highly effective for local disease. It should be made widely available as an alternative therapy, and may prove particularly useful in four situations: (1) patients with pre-existing cytopenia, since bone marrow suppression is a known side effect of ganciclovir; (2) patients who are poorly compliant with oral treatment; (3) patients unable to swallow pills or with poor gastrointestinal absorption; and (4) patients who have clinically failed to respond to oral medication. Initiating treatment with intraocular injection (for two weeks) in addition to oral valganciclovir may also be considered for “induction,” in order to most rapidly gain control of infection in patients when the disease is near the optic nerve or macula and is thus immediately sight-threatening.

Unfortunately, the availability of intraocular ganciclovir treatment in resource-poor settings has been extremely limited, and this situation will probably continue. Clinicians trained in intraocular injection are in short supply, and local regulations, such as in Thailand, may restrict this treatment to ophthalmologists. Also, intraocular injection is an invasive treatment with a low rate of serious complications (endophthalmitis, vitreous hemorrhage, retinal detachment, and cataract) [37,38]. Patient acceptance is another barrier and intraocular injection can be frightening to patients. Compliance may be especially difficult in patients who would benefit most—those with minor or no symptoms. As the capacity for diagnosis of CMV retinitis develops, a higher volume of services will be required, including the presentation of more patients with asymptomatic disease. The intraocular injection strategy will grow still less satisfactory. Providing a simple pill is far more realistic.

We believe that systemic treatment with oral valganciclovir should be used routinely as the primary treatment strategy because (1) systemic treatment of CMV retinitis reduces extraocular CMV disease [39]; (2) systemic treatment reduces mortality [40,41]; and (3) with only local treatment there is a 22%–35% incidence of new CMV retinitis in the untreated contralateral eye [37,38]. Intraocular ganciclovir as the primary treatment strategy is simply not medically adequate.

Valganciclovir is an essential drug for the treatment of CMV retinitis. Without this option most patients with CMV retinitis will never be treated.

## Consequences of Neglecting CMV Retinitis

We have unequivocally observed that CMV retinitis is causing blindness in a young population in developing countries (Table 2), even though the full scope of the problem remains to be defined.

**Table 2:**
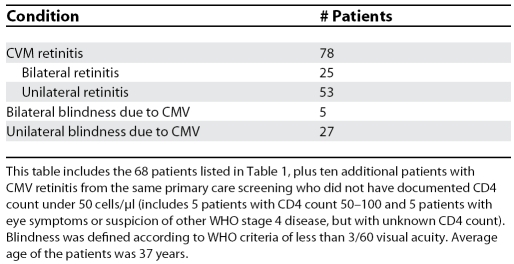
Blindness among 78 Patients with CMV Retinitis from Cambodia, Uganda, South Africa, Thailand, and Myanmar

The vision loss from CMV is commonly profound or total (Table 3), and permanent, which means that the patient will require near constant assistance from another individual and retain little or no capacity for independent existence. The stark reality is that conditions favorable to the development of CMV retinitis—patients with low CD4 counts in the absence of HAART—are most common in impoverished rural areas where profound blindness will have a devastating impact on the entire family, and often a fatal outcome for the patient. For the cohort of blind patients that survive, there looms the social, economic, and indirect personal health consequences of permanent profound blindness at a young age, coupled with HIV infection, a now treatable chronic disease.

**Table 3:**
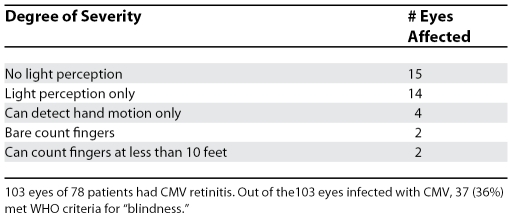
Severity of Vision Loss in 37 Eyes (of 32 Patients) “Blinded” by CMV Retinitis

Failure to diagnose CMV retinitis, and failure to treat affected patients with systemic anti-CMV medication, has an unfavorable impact on AIDS-related morbidity and mortality, because CMV is a systemic disease [42], and the extraocular forms of CMV, including colitis, esophagitis, encephalitis, radiculitis, pneumonitis, bone marrow suppression, and disseminated CMV infection, can be life-threatening. In the West, in the pre-HAART era, CMV infection was found at autopsy in over half of patients with AIDS [43,44,45], and CMV disease of the central nervous system (CNS) was found in about 10% of autopsy patients [46,47,48].

At least in patients with unexplained CNS disease, detection or exclusion of CMV retinitis has clear clinical diagnostic value: CNS disease in a patient with CMV retinitis is probably also due to CMV, while in patients free of retinitis, CNS symptoms are probably not from CMV infection [49,50]. Published studies report extraocular CMV disease as the primary cause of death in 1%–19% of patients with AIDS [2,43,51], and reduction in mortality has been observed with systemic treatment of CMV retinitis [40], even in patients failing HAART therapy [41].

Our experience in resource-poor settings is consistent with previous reports of morbidity and mortality from extraocular CMV disease. Between November 2001 and September 2006, MSF initiated antiretroviral therapy in 330 adult patients in two rural district hospitals in Northeast Thailand (Ban Laem District Hospital and Kuchinarai District Hospital), and 28 patients with visual symptoms were diagnosed with CMV retinitis (eye screening exams were not done). Six of the 28 patients with CMV retinitis also had disease clinically suggestive of extraocular CMV (one with ascending polyradiculopathy, two with encephalitis, two with esophagitis, and one with colitis). Four of these patients died.

Finally, experience in Cambodia, Thailand, and Myanmar shows that, at least in Southeast Asia, in the first several years, programs that offer HAART in resource-poor settings attract large numbers of patients with advanced disease and low CD4 counts—exactly those most vulnerable to CMV retinitis. CMV management becomes a pressing issue with the scale-up of HAART, and failure to address the problem of CMV retinitis will have negative consequences for the management of HIV/AIDS programs. Blindness is extremely distressing for the patient and family and also causes loss of faith in HIV treatment amongst health staff. In cases where loss of sight occurs without explanation after institution of HAART, patients may begin to associate HAART with blindness, and in the worst case scenario this may lead to reluctance to start taking this life-saving therapy.

## What Can Be Done?

Treatment of opportunistic infections is an integral part of HIV management at the primary care level, and treatment of CMV retinitis should be included. Patients with CMV retinitis are often too sick or lack adequate financial or social support to reach specialists [52]. In most places in the developing world there are too few ophthalmologists to manage the CMV problem, and like other specialists, they are concentrated in major cities, relatively inaccessible to rural locations and to the poor [53]. Ophthalmologists are often not highly motivated to care for patients with AIDS, and have other priorities, including a growing backlog of 45 million patients waiting for sight-restoring cataract surgery globally [54].

Two steps are critical:

1. **Diagnostic capacity must be expanded and decentralized to the primary care level.** HIV/AIDS programs that offer HAART should designate a clinician to be trained in retinal examination with the indirect ophthalmoscope. A sturdy portable indirect ophthalmoscope (Scan Optics, US$1050 complete with 28D lens; http://www.scanoptics.com.au/) is available, and has been field-tested in rural South Africa by an MSF HIV clinician who received a one-month pilot training course at the Aravind Eye Institute in India. This could be the first step in development of a simple training package for diagnosis and treatment of CMV retinitis at the primary care level, with identification and development of regional centers where training can take place. A similar step is being facilitated by MSF in Thailand (see Box 1).
2. **Valganciclovir must be made available and affordable.** Many HIV/AIDS programs in resource-poor settings are hesitant about investing resources to develop diagnostic capacity because treatment is unavailable or unaffordable. Making valganciclovir pills available for the treatment of CMV retinitis will provide powerful encouragement for HAART programs to address the problem of CMV.

**Box 1. Introducing a CMV Retinitis Diagnosis and Treatment Strategy in Thailand**

Thailand is one of few developing countries to provide HAART nationwide [55], treatment being available at 800 hospitals [56] and some health centers [57]. The national protocol for CMV retinitis diagnosis and treatment includes screening of patients with CD4 count below 100 cells/μl, induction therapy with intravenous ganciclovir, and maintenance therapy with either intravenous or intraocular ganciclovir [58]. In practice, however, diagnostic capacity only exists in specialist centers, and there is no allocation in the national budget for this disease, although it is planned for 2008.

In Kuchinarai District, Northeastern Thailand—the country's poorest region—200 people with HIV receive HAART through the district hospital in a project supported by MSF since 2002. Most patients first present with advanced disease, 17% having been diagnosed with CMV retinitis following visual symptoms. Regular screening by indirect ophthalmoscopy is being introduced, performed by two general medical practitioners following initial training at a tertiary referral hospital. Their initial training (one week only) could not provide complete skills, but was sufficient to create a referral system whereby patients with suspected CMV retinitis are referred to the tertiary center for confirmation of diagnosis and intraocular ganciclovir injection.

However, many patients live up to 25 km from the hospital and public transport in this district is lacking. For earlier diagnosis, we are introducing screening where the first patient contact often occurs—at the health station level. Screening is done by the hospital's general practitioners during monthly visits. Clinical management of CMV retinitis needs to be integrated into HIV care at both the district hospital and health station level, and availability of systemic treatment of CMV retinitis with oral valganciclovir would make this far more feasible.

Valganciclovir is available from Roche for transplant patients in Thailand, but the cost for treating CMV would be $US9,398 (Assumptions for price calculation: Induction phase of 900 mg twice daily for 21 days, followed by maintenance phase of 900 mg once daily for three months. A three month supply of a comparable off-patent product such as acyclovir is available for $US89 on the international market (median price). In negotiations with MSF's Access to Essential Medicines Campaign, Roche has offered $US1,800 for use in AIDS patients by nongovernmental organizations in sub-Saharan African countries and least developed countries. However, Thailand was excluded from this offer.

**(David Wilson and A. Kace Keiluhu)**

## Conclusions

The realistic place for managing CMV infection, like other opportunistic infections, is in the AIDS clinic by the AIDS doctors. Diagnosis and management of CMV infection should become part of routine care. Screening for CMV retinitis should be part of the initial evaluation of high-risk patients (at minimum all patients with CD4 counts below 50 cells/μl, and possibly other groups) when they first enter HIV care. The screening technique should be the same as in Western countries: study of the entire retina through a fully dilated pupil using an indirect ophthalmoscope.

Systemic treatment of CMV retinitis with oral valganciclovir should be standard care, just as it is in Western countries. Treatment with ganciclovir intraocular injection is easily affordable, but lack of trained clinicians skilled and available to perform intraocular injection restricts its use. We should aggressively move forward with training in essential skills and providing access to essential medicines.

Simple and effective management of CMV disease in resource-poor settings is a realistic goal, and one that has been overlooked in the scale-up of HIV treatment worldwide. Ongoing CMV-related mortality should no longer go unrecognized or be accepted as part of advanced HIV mortality. Patients should not be left vulnerable to blindness while clinicians are in the process of treating and controlling the underlying infection with HIV.

## Supporting Information

Alternative Language Summary S1 Thai Translation of the Summary by C. Klangburi and S. Kogram (71 KB PDF)

## Abbreviations

CMV: cytomegalovirus
CNS: central nervous system
HAART: highly active antiretroviral therapy
IRU: immune recovery uveitis
MSF: Médecins Sans Frontières
WHO: World Health Organization
