# Supplementary material for: Cytomegalovirus Retinitis: The Neglected Disease of the AIDS Pandemic
Source: PLoS Med. 2007 Dec 1;4(12):e334. doi: 10.1371/journal.pmed.0040334 (PMC2100142; doi:10.1371/journal.pmed.0040334)
Supplement: Supplementary file 1 [file pmed.0040334.sd001.pdf]

โรคจอประสาทตาอักเสบจากเชื้อ CMV: โรคจากภัยการแพร่ระบาดของโรคเอดส์ที่ถูกละเลย

David Heiden, Nathan Ford, David Wilson, William R. Rodriguez, Todd Margolis, Bart Janssens, Martha Bedelu, Nini Tun, Eric Goemaere, Peter Saranchuk, Kalpana Sabapathy, Frank Smithius, Emmanuel Luyirika, W. Lawrence Drew

บทคัดย่อ

โรคจอประสาทตาอักเสบจากเชื้อไซโตเมกะโลไวรัสหรือซีเอ็มวี (CMV) เป็นสาเหตุของการตาบอดที่พบบ่อยในผู้ป่วยและผู้ติดเชื้อเอชไอวี/เอดส์ในประเทศแถบตะวันตก ก่อนที่จะมีวิธีรักษาผู้ป่วยเอดส์โดยการให้ยาต้านไวรัสเอดส์ประสิทธิภาพสูงอันประกอบด้วยยาต้านไวรัสเอดส์ตั้งแต่3 ชนิดขึ้นไปหรือ HAART ทว่าสำหรับขอบเขตผลกระทบของโรคจอประสาทตาอักเสบจากเชื้อ CMV ต่อผู้ป่วยในประเทศด้อยพัฒนาในปัจจุบันนั้นยังไม่เป็นที่เข้าใจถ่องแท้ และเพื่อแก้ปัญหานี้คณะผู้เขียนจึงได้ทำการตรวจคัดกรองจอประสาทตาในผู้ป่วยและผู้ติดเชื้อเอชไอวี/เอดส์ ณ สถานีอนามัย (สถานพยาบาลขั้นปฐมภูมิ) ในประเทศแถบ Sub-Sahara (ประเทศที่ตั้งอยู่ทางใต้ของทะเลทรายซาฮาราในทวีปแอฟริกา) และในภูมิภาคเอเชียตะวันออกเฉียงใต้รวม 5 ประเทศ จากการตรวจผู้ป่วยจำนวน 325 รายที่มีผลการตรวจนับจำนวนเม็ดเลือดขาวชนิด CD 4 ต่ำกว่า 50 cells/ml พบว่ามีผู้ป่วย 68 รายหรือร้อยละ 20 ที่ได้รับการวินิจฉัยว่าเป็นโรคจอประสาทตาอักเสบจากเชื้อ CMV ซึ่งเป็นโรคที่โดยปกติมักไม่มีการตรวจพบมาก่อน และจากการศึกษาเพิ่มเติมพบว่า ดวงตาผู้ป่วยข้างที่ได้รับการวินิจฉัยว่าเป็นโรคจอประสาทตาอักเสบจากเชื้อ CMV นั้นมี 37 จาก 103 ราย (ร้อยละ 36) ที่บอดสนิทจากการติดเชื้อดังกล่าว ซึ่งการค้นพบเหล่านี้ล้วนสนับสนุนข้อพิสูจน์ที่ว่า โรคจอประสาทตาอักเสบจากเชื้อ CMV เป็นโรคฉวยโอกาสจากภัยการแพร่ระบาดของโรคเอดส์ที่ถูกละเลย และเป็นปัญหาทางคลีนิคหรือการตรวจรักษาอย่างมีนัยยะสำคัญยิ่ง ทั้งนี้ในการจัดการโรคจอประสาทตาอักเสบจากเชื้อ CMV ในพื้นที่ขาดแคลนให้ได้เป็นผลสำเร็จนั้นต้องอาศัยปัจจัย 2 ประการด้วยกันคือ การตรวจคัดกรองจอประสาทตาในผู้ป่วยกลุ่มเสี่ยงที่ม่านตาขยายอย่างเต็มที่ด้วยกล้องส่องตรวจจอประสาทตาเพื่อทำการวินิจฉัยโรคอย่างเป็นระบบ และ การรักษาด้วยการรับประทานยาวาลแกนซิโคลเวีย ซึ่งมีประสิทธิภาพในการรักษาทั้งโรคที่เกิดกับตาโดยตรงและโรคที่เกิดจากการแพร่กระจายของโรคไปทั่วร่างกาย ทว่ายาวาลแกนซิโคลเวียในขณะนี้มีราคาแพงมากจนผู้ป่วยไม่อาจเข้าถึงได้ ด้วยเป็นผลิตภัณฑ์ยาที่ได้รับสิทธิผูกขาดในการผลิตและจำหน่ายแต่ผู้เดียว จึงจำเป็นอย่างยิ่งที่จะต้องทำให้ยานี้มีแพร่หลายในราคาที่เหมาะสมและสามารถเข้าถึงได้

การริเริ่มยุทธศาสตร์การตรวจวินิจฉัยและการรักษาโรคจอประสาทตาอักเสบจากเชื้อ CMV ในประเทศไทย

ประเทศไทยเป็นหนึ่งในกลุ่มประเทศกำลังพัฒนาเพียงไม่กี่ประเทศที่ให้บริการการรักษาด้วยยาต้านไวรัสแบบ HAART แก่ประชาชน โดยให้บริการผ่านโรงพยาบาลกว่า 800 แห่งทั่วประเทศ ซึ่งนโยบายระดับชาตินี้ยังได้ให้บริการการตรวจวินิจฉัยและรักษา รวมถึงการตรวจคัดกรองผู้ป่วยเพื่อค้นหาเชื้อ CMV ในรายที่มีผลเม็ดเลือดขาวชนิด CD 4 ต่ำกว่า 100 การรักษาในระยะแรก ให้การรักษาด้วยยาแกนซิโคลเวียทางเลือดดำ และรักษาในระยะต่อเนื่องด้วยยาแกนซิโคลเวียทางหลอดเลือดดำหรือการฉีดยาแกนซิโคลเวียเข้าน้ำวุ้นลูกตาโดยตรง แต่ในทางปฏิบัติแล้วมีแต่ศูนย์เฉพาะทางเท่านั้นที่มีศักยภาพในการตรวจวินิจฉัยโรคนี้ได้ อีกทั้งยังไม่มีการจัดสรรงบประมาณของประเทศสำหรับการรักษาโรคจอประสาทตาอักเสบจากเชื้อ CMV แต่อย่างใด แต่ทั้งนี้ได้มีบรรจุอยู่ในแผนปี 2551 แล้ว

ในอำเภอกุฉินารายณ์ทางภาคตะวันออกเฉียงเหนือของประเทศไทย มีผู้ป่วยราว 200 รายที่ได้รับการรักษาด้วยยาต้านไวรัสแบบ HAART จากโรงพยาบาลประจำอำเภอตามโครงการที่องค์การหมอไร้พรมแดนได้ให้การสนับสนุนมาตั้งแต่ปี 2545 ผู้ป่วยส่วนใหญ่มักมาพบแพทย์ในระยะโรคที่เป็นมาก และมี 17 รายที่ตรวจวินิจฉัยพบว่าเป็นโรคจอประสาทตาอักเสบจากเชื้อ CMV พร้อมมีอาการทางสายตา ดังนั้นจึงได้ริเริ่มให้มีการตรวจคัดกรองด้วยกล้องส่องตรวจจอประสาทตาเป็นประจำ โดยแพทย์ประจำโรงพยาบาล 2 นายที่ได้รับการอบรมในเบื้องต้น ณ โรงพยาบาลระดับตติยภูมิซึ่งเป็นศูนย์ส่งต่อผู้ป่วย การอบรมในเบื้องต้นนี้ (เพียงระยะ 1 สัปดาห์) แม้ไม่อาจฝึกฝนทักษะที่สมบูรณ์ครบถ้วนได้ แต่ก็เพียงพอที่จะจัดทำระบบส่งต่อผู้ป่วยที่ต้องสงสัยว่าเป็นโรคจอประสาทตาอักเสบจากเชื้อ CMV ไปยังสถานพยาบาลระดับตติยภูมิเพื่อรับการวินิจฉัยยืนยันและการรักษาด้วยการฉีดยาแกนซิโคลเวียเข้าน้ำวุ้นลูกตาต่อไป

ทว่าผู้ป่วยจำนวนมากในอำเภอกุฉินารายณ์อาศัยอยู่ห่างไกลจากโรงพยาบาลประจำอำเภอถึง 25 กิโลเมตร อีกทั้งยังขาดแคลนระบบบริการขนส่งสาธารณะ ดังนั้นในการตรวจวินิจฉัยเบื้องต้นจึงใช้วิธีตรวจคัดกรองแก่ผู้ป่วย ณ ที่ที่ผู้ป่วยมักมาพบแพทย์เป็นครั้งแรก นั่นคือ สถานีอนามัย โดยมีแพทย์จากโรงพยาบาลเดินทางมาตรวจเยี่ยมเป็นประจำทุกเดือน ด้วยเหตุนี้จึงต้องมีการผสานการจัดการทางคลินิคสำหรับโรคจอประสาทตาอักเสบจากเชื้อ CMV กับการรักษาโรคเอดส์ทั้งในโรงพยาบาลระดับอำเภอและสถานพยาบาลในชุมชน และเมื่อรวมกับการให้บริการการรักษาโรคจอประสาทตาอักเสบจากเชื้อ CMV ด้วยยาวาลแกนซิโคลเวียอย่างเป็นระบบแล้ว ย่อมช่วยให้โรคนี้มีหนทางรักษาได้มากยิ่งขึ้น

ยาวาลแกนซิโคลเวียจัดจำหน่ายโดยบริษัท Roche ใช้สำหรับผู้ป่วยที่ได้รับการผ่าตัดปลูกถ่ายอวัยวะในประเทศไทย แต่ในการใช้ยานี้เพื่อรักษาโรคจากเชื้อ CMV มีต้นทุนสูงถึง 9,398 เหรียญสหรัฐ ทั้งนี้จากการเจรจาต่อรองราคาโดยฝ่ายรณรงค์เพื่อการเข้าถึงยาจำเป็นขององค์การหมอไร้พรมแดน บริษัท Roche ได้เสนอราคาที่ 1,800 เหรียญสหรัฐแก่องค์กรเอกชนต่างๆ ที่ต้องการใช้ยานี้รักษาผู้ป่วยโรคเอดส์ในประเทศแถบ Sub-Sahara ในทวีปแอฟริกาและประเทศที่ด้อยพัฒนาที่สุด แต่ไม่รวมประเทศไทย

ขอขอบคุณ:
ผู้ช่วยศาสตราจารย์นายแพทย์ธนภัทร รัตนภากร ภาควิชาจักษุวิทยา คณะแพทยศาสตร์ มหาวิทยาลัยขอนแก่น ประเทศไทย ผู้ให้การอบรมและติดตามผลแก่เจ้าหน้าที่แพทย์เวชปฏิบัติทั่วไปในอำเภอกุฉินารายณ์
